# Effect of *Burkholderia ambifaria* LK-P4 inoculation on the plant growth characteristics, metabolism, and pharmacological activity of *Anoectochilus roxburghii*

**DOI:** 10.3389/fpls.2022.1043042

**Published:** 2022-12-02

**Authors:** Juanying Wang, Hanyu Zhao, Ting Chen, Wenxiong Lin, Sheng Lin

**Affiliations:** ^1^ Fujian Provincial Key Laboratory of Agroecological Processing and Safety Monitoring, College of Life Sciences, Fujian Agriculture and Forestry University, Fuzhou, China; ^2^ Key Laboratory of Plant Resources Conservation and Germplasm Innovation in Mountainous Region (Ministry of Education), Guizhou Key Lab of Agro-bioengineering, Institute of Agro-bioengineering/College of Life Science, Guizhou University, Guiyang, China; ^3^ Fujian Provincial High Education Key Laboratory of Crop Physiology and Molecular Ecology, College of Agronomy, Fujian Agriculture and Forestry University, Fuzhou, China

**Keywords:** *Anoectochilus roxburghii*, PGPB inoculation, morphology, bioactive compounds, metabolomics

## Abstract

**Background:**

Plant growth-promoting bacteria (PGPB) represents a common biological fertilizer with remarkable effect in improving crop production and environmental friendliness.

**Methods:**

In the present work, we presented a detailed characterization of plant morphology and physiology, metabolism, and pharmacological activity of *A. roxburghii* between *Burkholderia* ambifaria LK-P4 inoculation and un-inoculation (CK) treatment by routine analytical techniques (include microscopy and enzymatic activity assays and so on) coupled with metabolomics approaches.

**Results:**

Morphological and physiological results showedthat the P4 bacteria could significantly increase plant stomatal density, freshweight, survival rate,and the content of total flavonoids in leaves but reducethe amount of free amino acid. Furthermore, metabolite data showed that fatty acids (linoleic acid, linolenic acid, stearic acid) and active substance (kyotorphin and piceatannol) were specifically up-regulated in P4 inoculation. It was also demonstrated that the differential metabolites were involved in citrate cycle, glyoxylate and dicarboxylate metabolism, and biosynthesis of unsaturated fatty acids pathway. In addition, pharmacological efficacy found that *A. roxburghii* under P4 inoculation can significantly decrease (p < 0.05) blood glucose levels and protect the organs of mice with similar effect of Glibenclamide tablets.

**Conlusion:**

Overall, it can be seen that the exogenous P4 bacteria can promote the growth and increase content of special metabolites in *A. roxburghii*. This study provided theoretical basis and supported for the high-yield and high-quality bionic cultivation of *A. roxburghii*.

## Introduction

*Anoectochilus roxburghii*, a rare and valued medicinal plant that belongs to family *Orchidaceae*, is mainly distributed in Fujian, Guangxi, and Zhejiang and is known as “king medicine” and “golden grass” in China. It contains polysaccharides, alkaloids, flavonoids, organic acids, amino acids, and trace elements, which can be widely used in medicine, healthcare, drinking products, and many other areas (Shao et al., 2014b; Yu et al., 2017; Wang et al., 2020). Pharmacological studies have revealed that this plant has been widely used as a folk medicine to treat diabetes, tumors, cancers, hepatitis, hyperliposis, and cardiovascular disease (Shao et al., 2014a; Tang et al., 2018).

However, wild *A. roxburghii* is now facing extinction as a result of specific seed germination conditions symbiotic with fungi, slow growth rate, the heavy demand of its wild resources, and destruction of its habitat. Thus, using the tissue culture technology combined with bionic cultivation (transplanting the tissue culture seedlings to the natural environment cultivation) instead of being harvested from wild populations was considered as a desirable approach to meet the growing demands for this plant, as well as to maintain its quality. In this process, due to the complexity of the environment, the effectiveness of the method remained elusive. In the last decade, numerous scholars have carried out studies on the cultivation substrate, transplanting month, planting density, light, and other aspects to improve the plant productivity of *A. roxburghii* (Yu and Shi, 2016; Huang et al., 2017; Niu et al., 2018). However, little is known about the application of the rhizosphere microbiota, particularly plant growth-promoting bacteria (PGPB), for the cultivation of *A. roxburghii*. Therefore, it has become a priority to explore new ways for microorganisms to maintain the sustainable development of the *A. roxburghii* industry.

The rhizosphere microbiome, defined as “second genomics” of plants, is crucial to plant development, health, and productivity through secreting diverse signaling molecules, participating in various biological processes, and regulating carbon and nutrient cycling (Rout and Southworth, 2013; Compant et al., 2019; Jin et al., 2022). The use of microbes to deal with plant growth problems presently referred to an environment-friendly and biological control approach without any side effects. Inoculation with beneficial bacteria, especially PGPB, has advantages and broad application prospects in sustainable agriculture, which has received considerable attention (Dobbelaere et al., 2003; Souza et al., 2015). There bacteria often designated as PGPB, including *Rhizobium*, *Bradyrhizobium*, *Azospirillum*, *Pseudomonas*, *Bacillus*, *Burkholderia*, *Herbaspirillum*, and *Streptomyces*, were successfully shown to have a profound effect on improving plant production and productivity (Bashan et al., 2014; Santos et al., 2017). A series of research progress showed that inoculation with *Azospirillum* can promote elongation of root, development of lateral and adventitious roots, and branching of root hairs (Dobbelaere et al., 1999; Creus et al., 2005; Molina-Favero et al., 2008). The underlying mechanisms involving inoculation were very complex, both direct and indirect. Examples of direct plant growth promotion included nutrient acquisition, such as nitrogen fixation and phosphorus solubilization, and phytohormone secretion, such as auxin and release of volatile organic compounds (VOCs), to inhibit pathogen. Indirect benefits occurring in the rhizosphere have been documented such as induction of systemic resistance (ISR) and competition for nutrients and niches (Bashan and De-Bashan, 2010; Pii et al., 2015; Gouda et al., 2018). In our previous screening, it was found that some PGPB had diverse plant growth promotion traits and the ability to enhance the growth of *Radix pseudostellariae* L. and *A. roxburghii* L. (Wu et al., 2019; Yang et al., 2022). However, the mechanisms of PGPB that ensure a better adaptation to terrestrial life from tissue culture of *A. roxburghii* remained unknown, which encouraged us to further explore the issue.

Metabolic profiling is the most important approach for plants to understand chemical diversity. For medicinal plants, these metabolic compounds that mainly consisted of flavonoids, alkaloids, polysaccharides, and low-molecular-weight organic compounds not only were the key determinants of medicine efficacy, but also functioned in many facets of plant physiology. While genes were able to control the production of secondary metabolites, biotic and abiotic environments such as climate and edaphic factors or plant or soil microbes have been shown to play a key role in their specific expression (Giweli et al., 2013; Sampaio et al., 2016). As mentioned before, the ISR system was closely related to the metabolism of plants. Precisely, after inoculation with bacteria, plants will trigger jasmonic acid and ethylene signaling to protect themselves from certain pathogens, fungi, and viruses (Van Loon, 2007). Thus, microbial activity in soil can greatly influence plants. On the other hand, through roots, plants were able to supply nutrients or establish a habitat for their rhizosphere microflora by the release of up to 40% photosynthetically fixed carbon (Bais et al., 2006; Rudrappa et al., 2008). The most successful adaptation of plant cannot divorce from the interaction between plant metabolites and microorganisms.

The aim of this study was to evaluate the performance of *B. ambifaria* LK-P4 for the plant morphology, physiology, and metabolism of *A. roxburghii*. It will provide information that will increase our understanding of possible plant-promoting mechanisms and new management approach for the cultivation of *A. roxburghii.* Furthermore, we evaluated pharmacological activity through examining blood glucose and serum indexes of diabetic mice fed with *A. roxburghii* under different treatments, which could shed light on the usability and safety of this medicinal plant.

## Materials and methods

### Bacterial strains

*B. ambifaria* LK-P4 was selected from the results of the previous screenings (Wu et al., 2019; Yang et al., 2022) and was obtained from Fujian Provincial Key Laboratory of Agroecological Processing and Safety Monitoring, College of Life Sciences, Fujian Agriculture and Forestry University, which was originally isolated from the rhizosphere soil of the Chinese herbal medicinal plant *R. pseudostellariae*. It was grown in Luria-Bertani (LB) medium under orbital shaking at 200 rpm and 37°C for 12 h, collected and washed three times with double-distilled water, and then resuspended to the final concentration of 1×10^8^ cfu/ml (OD_600_ = 0.5) in sterile double-distilled water to be used as inoculants.

### Rhizobox experiment

*A. roxburghii* tissue culture cultivar used in the trial was “Fujian Xiaoyuanye” from Fujian NaHoe Agricultural Technology Co., Ltd. After acclimatization for a week, healthy plantlets with a consistent size were soaked in a 1,000-fold dilution of carbendazim for 5 min and transplanted in a pot (8 × 5.5 × 6.5 cm) filled with 200 g of sterilized cultivated substrate soil. The experimental setup included two treatments with 12 replicate pots (three plantlets per pot) per treatment in a complete randomized design: plants inoculated with P4 plus a control treatment with water inoculation. At 2 weeks after plantlet transplant, the inoculation with 600 μl of bacterial cells was carried out three times onto *A. roxburghii* plants at the rooting site. Two treatments were placed in a growth chamber at 16–23°C with a day length of 12 h, and light intensity was set at 1,000 lux.

### Effect of bacterial inoculation on the morphology and physiology of *A. roxburghii*

Plant growth parameters including root activity, enzyme activity, kinsenoside, and leaf anatomical characteristics were measured during the physiological maturity stage. Briefly, root activity was analyzed by the triphenyl tetrazolium chloride (TTC) method (Zhang et al., 2013); 0.5 g fresh root was immersed in 10 ml of an equally mixed solution of 0.4% TTC and phosphate buffer, and kept in the dark at 37°C for 1 h. Subsequently, 2 ml of 1 mol/L H_2_SO_4_ was added to stop the reaction with the root and then extracted with ethyl acetate. The absorbance of the extract at 485 nm was recorded. The leaf enzyme activities including peroxidase (POD), superoxide dismutase (SOD), and catalase (CAT) were determined as described elsewhere (Mahdavikia et al., 2017). The extraction procedures for kinsenoside followed the method described by Cheng et al. (2015) with slight modifications. High-performance liquid chromatography (HPLC) with an Agilent ZORBAX SB-Aq column (4.6 mm × 250 mm, 5 μm) and a SPOD detector was used to calculate the yield of kinsenoside, and the mobile phase consisted of acetonitrile and water (75:25 v/v) run in isocratic mode at a flow rate of 1 ml/min. *A. roxburghii* stem structure, leaf stomata, and mesophyll cells were examined under an upright fluorescence electron microscope (Nikon Eclipse Ni-U).

During the experimental period, root, stem, and leaf tissue of *A. roxburghii* were also collected at different growth stages for bioactive compound analysis. In detail, a portion of 0.3 g of *A. roxburghii* sample was extracted with 10 ml of water at 100°C for 30 min and centrifuged (10,000 rpm, 5 min) for total polysaccharide solution. The content of polysaccharide in the extract was determined by the phenol-sulfuric acid method at 485 nm (Jiang et al., 2010). A modified version of the NaNO_2_-Al(NO_3_)_3_-NaOH method (Zhu et al., 2009) was used to quantify total flavonoids, with Rutin (Aladdin) as the standard. The absorbance of the solution at 510 nm was measured using a spectrophotometer. Polyphenols were determined by the Folin–Ciocalteu method (Costa et al., 2016). The absorbance of the solution was measured at 760 nm, and gallic acid (Aladdin) was used as the standard. The amino acid in *A. roxburghii* was assayed by the method described by Qin (2013).

### Metabolite extraction and detection

Prior to metabolite extraction, previously frozen plant tissue (collected after 6 months of bacteria inoculation) in liquid nitrogen was ground to a fine powder. Metabolites were extracted from *A. roxburghii* tissue using a method adopted from Weckwerth et al. (2004). Briefly, 4.5 ml of ice-cold extraction solution (methanol:water = 3:1, v:v) was added to 0.5 g of powder sample, thoroughly mixed with 10 μl of ribitol (2 mg/ml), kept in an ice-cold ultrasound bath for 30 min to disassociate metabolites, and then centrifuged at 10,000 *g* for 10 min at 4°C. Supernatant (200 μl) was dried and dissolved in 10 μl of methoxamine hydrochloride (20 mg/ml pyridine) and incubated at 30°C for 90 min with continuous shaking. Then, 80 μl of *N*-methyl-*N*-trimethylsilyl-trifluoroacetamide (MSTFA) was added to derivatize polar functional groups at 37°C for 30 min. The derivatized samples were performed on gas chromatography tandem time-of-flight mass spectrometry (GC-TOF-MS, Agilent 7890) metabolomic workflows equipped with a capillary column (Agilent DB-5MS) and a chromatographic column (30 m × 250 μm × 0.25 μm). The injection volume was 1 μl. The temperatures of the injector, transfer line, and ion source were set as 280°C, 280°C, and 250°C, respectively. After a 1-min hold at 50°C, the column oven temperature was programmed to increase to 310°C at 10°C/min and then held for 8 min.

### Pharmacological evaluation of *A. roxburghii* in a diabetic mouse model

Healthy male ICR (Institute of Cancer Research) mice certified pathogen-free and weighing 18–22 g were collected from Beijing Weitong Lihua Laboratory Animal Technology Co., Ltd., Beijing, China. The mice were placed in cages and acclimatized at 22°C ± 2°C and 45%–55% relative humidity (RH) with free access to water and food in the departmental animal house. The mice were exposed to a 12-h light/dark cycle.

Mice were assigned to seven different groups (*n* = 10): Group I (NM), control group (distilled water; 0.5 ml/kg, b.w.; i.p.); Group II, the DM model (1% alloxan; 120 mg/kg, b.w.; i.p. + 80 mg/kg, b.w.; i.p.) in which diabetic mice with blood glucose values >16.8 mmol/L after 72 h of the first administration were selected; Group III (DM-G), standard group (Model + Glibenclamide tablets; 20 mg/kg, b.w.; i.p.); Group IV (DM-PH), *A. roxburghii* purchased from pharmacy treatment (Model + *A. roxburghii* purchased from pharmacy; 1.25 g/kg, b.w.; i.p.); Group V (DM-CK), *A. roxburghii* in CK treatment (Model + *A. roxburghii* in CK; 1.25 g/kg, b.w.; i.p.); Group VI (DM-P4), *A. roxburghii* in P4 inoculation treatment (Model + *A. roxburghii* in P4 inoculation; 1.25 g/kg, b.w.; i.p.); and Group VII (DM-TC), *A. roxburghii* in tissue culture treatment (Model+ *A. roxburghii* in tissue culture shown in **Table 1**). All the drugs and powder of *A. roxburghii* were freshly prepared in distilled water before administration by gavage in mice. The duration (14 consecutive days) and dose selection were based on previous research studies with some modifications (Song et al., 2007; Cui et al., 2013; Deng et al., 2014). The body weight of mice was evaluated every 2 days during different treatments. After 14 days of drug treatment, mice (12 h after the last dose of drugs) were subjected to blood glucose analysis. At the same time, the mouse serum was collected for triglyceride (TG), total cholesterol (TC), high-density lipoprotein (HDL), low-density lipoprotein (LDL), malondialdehyde (MDA), superoxide dismutase (SOD), and catalase (CAT) analysis. The weight of nine organs and tissues, namely, heart, liver, spleen, lung, kidney, thymus, pancreas, gonad, and femur, was analyzed after dissection.

**Table 1:** Grouping and feeding of experimental mice.

Group	(*n*)	Treatment	Drug type and dose
I	10	NM	0.5 ml of distilled water
II	10	DM	1% alloxan (120 mg/kg, b.w.; i.p. + 80 mg/kg, b.w.; i.p.)
III	10	DM-G	1% alloxan (120 mg/kg, b.w.; i.p. + 80 mg/kg, b.w.; i.p.) + Glibenclamide tablets (20 mg/kg, b.w.; i.p.)
IV	10	DM-PH	1% alloxan (120 mg/kg, b.w.; i.p. + 80 mg/kg, b.w.; i.p.) + *A. roxburghii* purchased from pharmacy (20 mg/kg, b.w.; i.p.)
V	10	DM-CK	1% alloxan (120 mg/kg, b.w.; i.p. + 80 mg/kg, b.w.; i.p.) + *A. roxburghii* in CK (20 mg/kg, b.w.; i.p.)
VI	10	DM-P4	1% alloxan (120 mg/kg, b.w.; i.p. + 80 mg/kg, b.w.; i.p.) + *A. roxburghii* in P4 inoculation (20 mg/kg, b.w.; i.p.)
VII	10	DM-TC	1% alloxan (120 mg/kg, b.w.; i.p. + 80 mg/kg, b.w.; i.p.) + *A. roxburghii* in tissue culture (20 mg/kg, b.w.; i.p.)

DM, Diabetic Model [1% alloxan (120 mg/kg, b.w. + 80 mg/kg, b.w.)]; b.w., Body weight; i.p., Intraperitoneal; (n), Number of animals per group.

### Statistical analysis

ChromaTOF software was used to perform peak extraction, baseline correction, and peak integration analysis on mass spectrometry data. Substance qualitative analysis was performed using the LECO-Fiehn Rtx5 database. Finally, features with a detection rate below 50% or a relative standard deviation (RSD) >30% should be removed from the subsequent analysis. The resulting three-dimensional data involving the peak number, sample name, and normalized peak area were fed to R package metaX for principal component analysis (PCA) (Wen et al., 2017). The VIP (variable importance in the projection) values exceeding 1.0 were first selected as changed metabolites. In step 2, the remaining variables were then assessed by Student’s *t*-test (*Q*-value >0.05) and variables were discarded between two comparison groups. In addition, commercial databases including KEGG (http://www.genome.jp/kegg/) and MetaboAnalyst (http://www.metaboanalyst.ca/) were utilized to search for the pathways of metabolites. Differences between the treatments were calculated and statistically analyzed using analysis of variance (ANOVA) and Tukey’s test (*p* < 0.05). The Statistical Package for GraphPad Prism version 7 and the Data Processing System (DPS) version 7.05 were used for statistical analysis.

## Results

### The growth traits and enzyme activities of *A. roxburghii*

**Figure 1:**
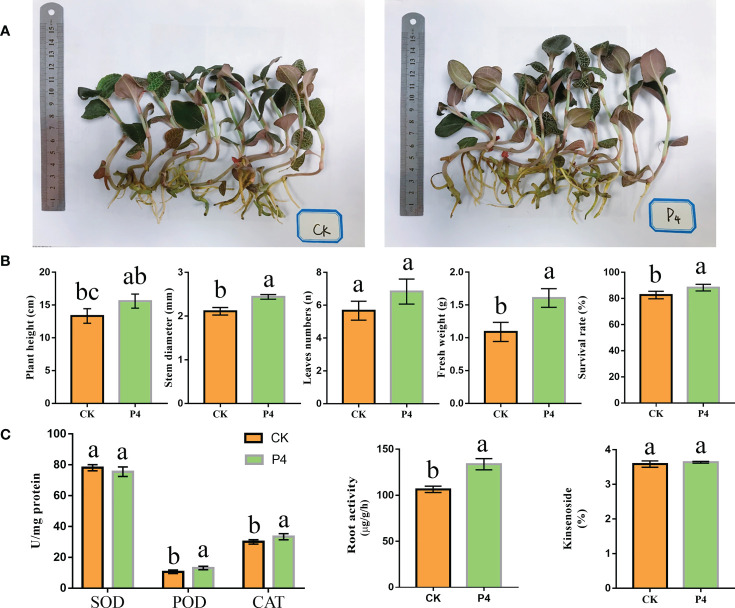
The growth traits of *A. roxburghii*. **(A)** Photograph of above- and belowground of *A. roxburghii* under different treatments. **(B)** The morphological traits of *A. roxburghii*. **(C)** Enzyme activity (including SOD, POD, and CAT), root activity, and kinsenoside of *A. roxburghii*. Different letters in columns indicate significant difference determined by Tukey’s test (*p* < 0.05; *n* = 3).

The P4 inoculation affected the morphological traits of *A. roxburghii* (**Figure 1A**). Plant stem diameter, fresh weight, and survival rate were significantly higher in the P4 treatment than in CK (**Figure 1B**). However, there was no significant difference in leaf numbers between P4 and CK treatments. As shown in **Figure 1C**, compared to CK treatment, P4 increased root activity by 25%. After the 6-month treatment, the SOD, POD, and CAT activities were different between CK and P4. The POD and CAT activity in the P4 treatment were 13.07 U/mg protein and 33.27 U/mg protein, respectively. The activities of these enzymes were significantly higher in the P4 treatment than in CK (**Figure 1C**). SOD activity did not differ significantly between the P4 treatment and CK. HPLC analysis showed that P4 inoculation had no significant effect on the content of kinsenoside.

**Figure 2:**
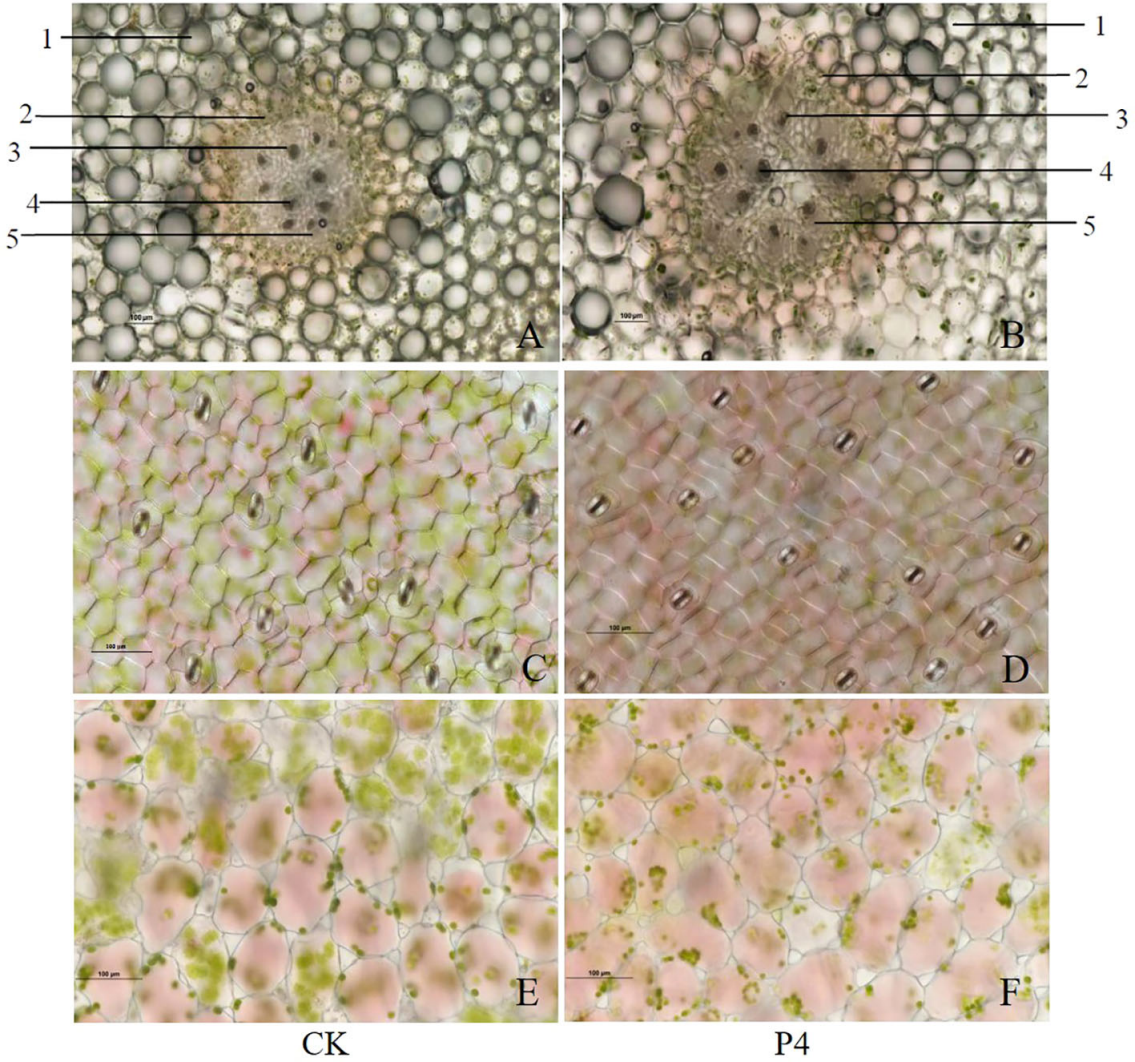
Morphological characteristics of stem **(A, B)**, anatomical structure of lower epidermis **(C, D)**, and mesophyll cells **(E, F)** in the leaf of *A. roxburghii*. **(A, C, E)** represented *A. roxburghii* in CK treatment; **(B, D, F)** represented *A. roxburghii* in P4 inoculation. 1 represents cortex, 2 represents endodermis, 3 represents phloem, 4 represents xylem, and 5 represents marrow.

Transversal section of the stem presented a primary anatomical structure: cortex, endodermis, phloem, xylem, and pith (**Figures 2A, B**). Compared with CK, the myeloid cells were significantly enlarged in P4 treatment. The stela scattered with nine vascular bundles of CK accounts for about 30% of the visual field, which was lower than those (stela scattered with 10 vascular bundles accounts for about 50% of the visual field) in the P4 treatment. The lower epidermal cells of the leaves of *A. roxburghii* were honeycomb-shaped, polygonal, and regular-shaped as can be seen in **Figures 2C, D**. However, cells were longer and narrower in CK than those in P4 plants. Stomatal frequency (the average number of stomata) was significantly higher in the P4 treatment (16) than in the CK (12) under the same 20× lens field. The results showed that the mesophyll cells in P4 were oval-like and tightly arranged, and chloroplasts gathered at the intercellular junctions (**Figures 2E, F**).

### Bioactive compound content of *A. roxburghii* under different treatments

**Figure 3:**
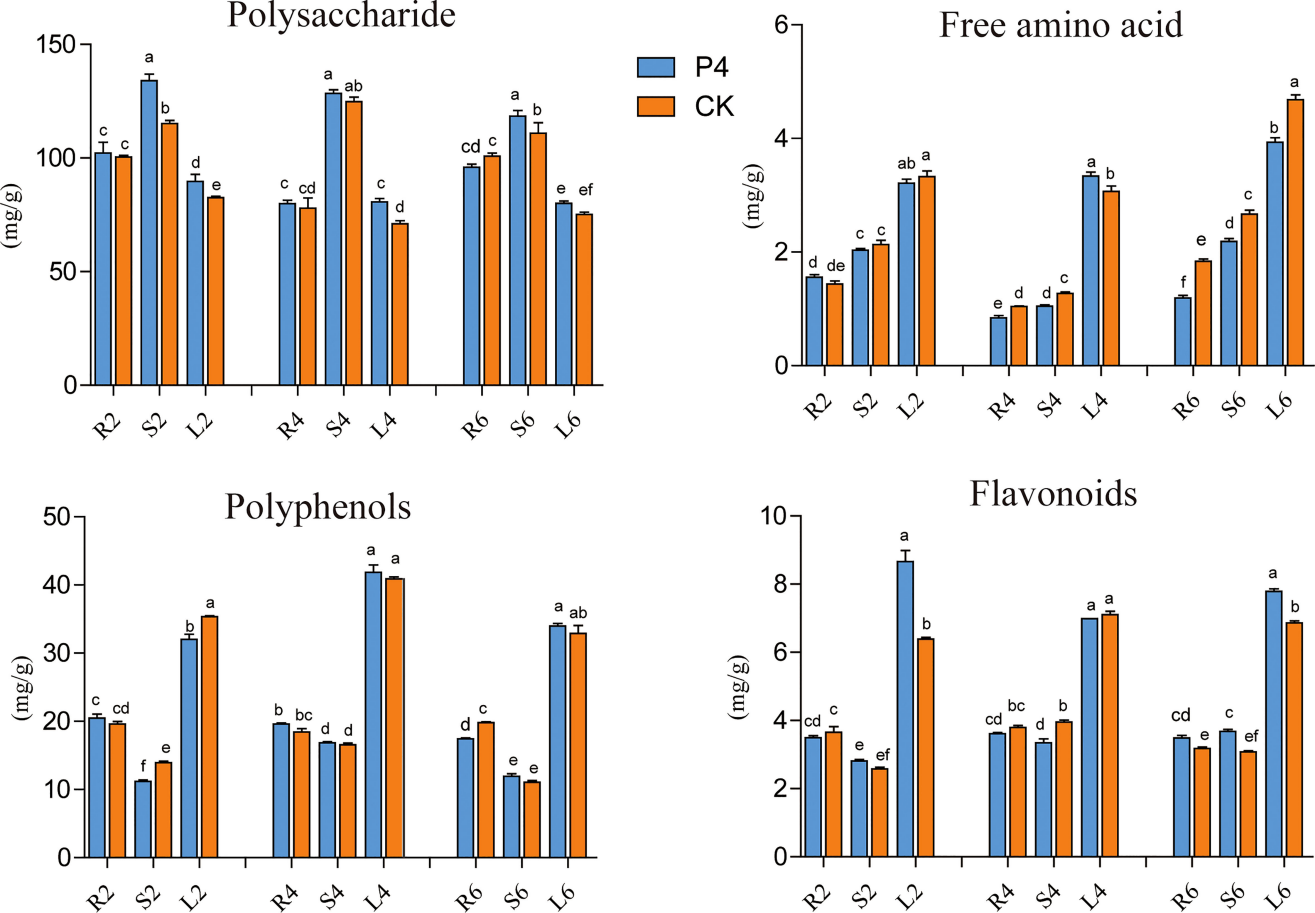
Dynamic changes of bioactive compounds in different tissues of *A. roxburghii*. R, Root, S, Stem, L, Leaf; 2, 4, and 6 represented different months after different treatments. Different letters in columns indicate significant difference determined by Tukey’s test (*p* < 0.05; *n* = 3).

The impact of inoculation of the bacterium on plant bioactive compounds (including polysaccharide, flavonoids, polyphenols and amino acids) was evaluated every 2 months after bacteria inoculation. In general, except for polysaccharides, the content of flavonoids, polyphenols, and free amino acids in leaves was higher than that in stems and roots (**Figure 3**). When referring to a specific tissue, the analysis showed that the bioactive compounds fluctuated in different plant growth stages. However, the significant positive effect of bacteria inoculation was also observed in terms of root, stem, and leaf tissue (**Figure 3**). The content of polysaccharides in the stems of P4 treatment in different sampling months was higher than that of CK, which was 1.16, 1.03, and 1.07 times that of CK, respectively. A similar appearance was observed in the case of leaves. Furthermore, the P4 bacteria also had a great influence on the total flavonoid content. Precisely at 6 months’ sampling time, the total flavonoid content in all tissue was higher in P4 than in CK. It was worth mentioning that the free amino acids of *A. roxburghii* in the P4 treatment were significantly lower than those in CK especially under 6 months. These results showed that bacterial inoculation strongly affected the content of bioactive compounds in *A. roxburghii*.

### Metabolic profile of plant compounds

**Figure 4:**
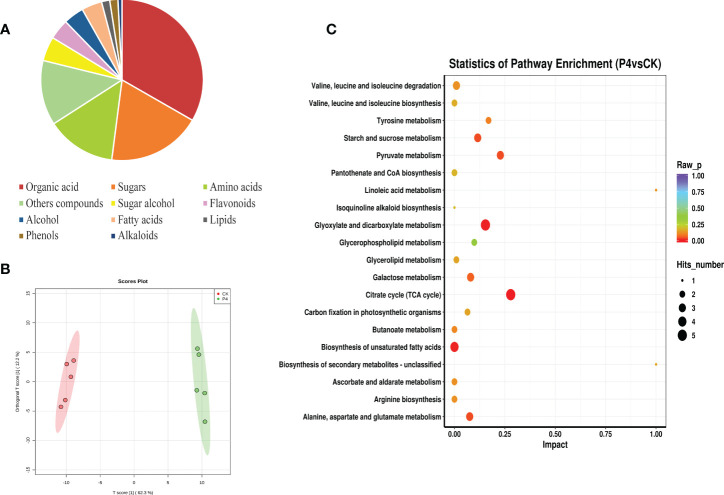
Metabolic profile of plant compounds. **(A)** Classification and **(B)** PLS-DA score plot of metabolite. **(C)** Influence factors of metabolic pathway.

The metabolomic identification approach by GC-TOF-MS allowed us to putatively annotate a total of 123 compounds, which could be classified into 11 chemical classes such as organic acid (33%), sugars (19%), amino acids (14%), sugar alcohol (5%), flavonoids (4%), and fatty acids (4%), among others (**Figure 4A**). These multivariate data obtained from the primary and secondary metabolite of *A. roxburghii* were subjected to PCA to investigate the differences in metabolic profiles among CK and P4 samples. The results (**Figure 4B**) showed obvious clustering in each group and significant differences in metabolite in PC1 levels between the two groups.

To investigate the relationships between the various metabolites detected in CK and P4, hierarchical cluster analysis (HCA) was performed using Pearson’s correlation results on the datasets (**Figure 4C**). Overall, a total of 19 compounds with a standard of variable important in projection (VIP) of ≥1 and a *p*-value of ≤0.05 were identified by evaluation of all of the detected metabolites and characterized by a strong up- or downregulation in plant according to the inoculation treatment (**Figure 4C**; **Table 2**). Focusing on nine compounds, specifically upregulated metabolites in the plant inoculated with the P4 strain, the results were shown to be mostly represented by fatty acids (linoleic acid, linolenic acid, stearic acid), amino acid (kyotorphin), and phenol (piceatannol). Considering the downregulated metabolites (**Figure 4C**), 10 compounds were altered in the plant, which were mainly organic acid compounds (including tropic acid, elaidic acid, 3-hydroxypropionic acid, and glucoheptonic acid) together with the amino acids. According to the Kyoto Encyclopedia of Genes and Genomes (KEGG) pathway database, the main enriched metabolic processes were involved in citrate cycle, glyoxylate and dicarboxylate metabolism, and biosynthesis of unsaturated fatty acids in CK and P4 (**Figure 4D**). Differential metabolites including succinic acid, maleic acid, and L-malic acid were target metabolites in citrate cycle metabolic pathways. The changes in content of these metabolites including linoleic acid and linolenic acid may play an important role in the biosynthesis of unsaturated fatty acids.

**Table 2:** Identification results of important metabolites.

Metabolite	RT (min)	VIP	*p*-value	*Q*-value	Log_2_FC
Tropic acid	14.741	1.122	0.00092	0.0019	−0.7510
Elaidic acid	21.052	1.160	0.00033	0.0007	−0.6553
Phytosphingosine	24.165	1.144	0.00025	0.0006	−0.6175
Alanine	8.071	1.161	0.00021	0.0005	−0.5996
3-Hydroxypropionic acid	8.626	1.183	0.00017	0.0005	−0.5407
Stearic acid	21.277	1.140	0.00030	0.0007	−0.4653
Cycloleucine	9.5850	1.136	0.00065	0.0014	−0.4151
D-talose	17.706	1.133	0.00044	0.0010	−0.3953
Glucoheptonic acid	21.457	1.139	0.00029	0.0007	−0.3924
Trehalose	25.137	1.174	0.00010	0.0003	−0.2487
Linoleic acid	21.000	1.126	0.00069	0.0014	0.3522
3-hydroxypalmitic acid	21.098	1.167	0.00021	0.0005	0.4055
Piceatannol	23.549	1.081	0.00154	0.0031	0.4629
Threitol	13.234	1.089	0.00143	0.0029	0.4943
Linolenic acid	21.058	1.176	0.00014	0.0004	0.5559
Conduritol b epoxide	18.576	1.175	0.00017	0.0005	0.5573
3-Hexenedioic acid	13.602	1.147	0.00064	0.0014	0.5802
Thymidine	15.155	1.105	0.00160	0.0032	0.5843
Kyotorphin	23.774	1.179	0.00014	0.0004	0.8647

### Pharmacological evaluation of *A. roxburghii* after P4 inoculation

**Figure 5:**
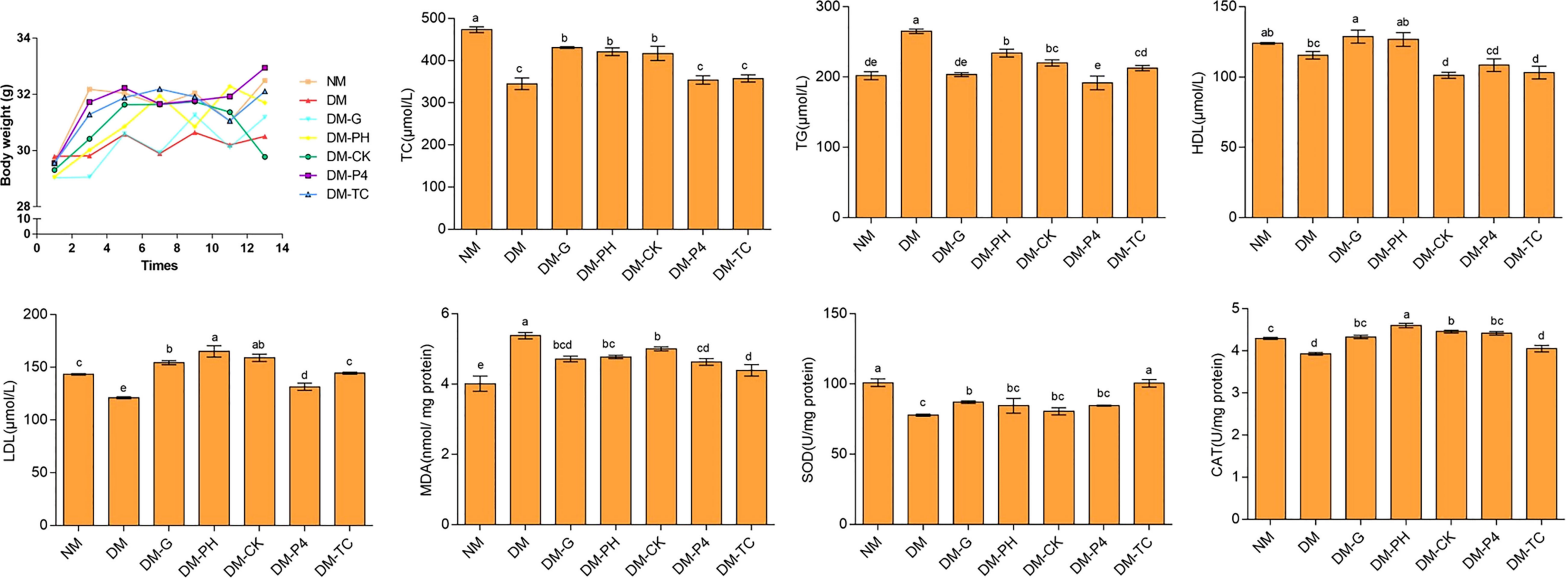
Body weight and serum biochemical indexes of mice under different treatments. Different letters in columns indicate significant difference determined by Tukey’s test (*p* < 0.05; *n* = 10).

Polydipsia and polyuria significantly appeared in the model with 1% alloxan administration as compared to the normal group (NM) and were sustained throughout the feeding period in the DM group. The situation improved to a certain extent after the mice were treated by Glibenclamide tablets or *A. roxburghii*. Changes in the body weight of mice during feeding are shown in **Figure 5**. Overall, compared with other groups, the body weight of the diabetic model (DM) mice was at a lower level. Long-term administration of Glibenclamide tablets increased cumulative body weight by nearly 7% at the end of the study (day 14), but still did not reach the weight levels of NM mice. Analysis of the data every 2 days of experiment demonstrated that long-term *A. roxburghii* treatment maintained the body weight of DM-TC, DM-P4, DM-PH, and DM-CK at the same level as NM mice. There was no significant change in body weight in the four treatments (DM-TC, DM-P4, DM-PH, and DM-CK), suggesting that *A. roxburghii* in P4 inoculation may not alter nutrient partitioning. Four indicators—triglyceride (TG), total cholesterol (TC), high-density lipoprotein (HDL), and low-density lipoprotein (LDL)—in mouse serum were used to judge the blood lipid metabolism disorder of diabetic mice (**Figure 5**). It was found that compared with NM mice, DM mice had a 24% and 16% decrease in TC and LDL, respectively, and a 21% increase in TG. The TG content of mice treated with DM-G, DM-P4, and DM-TC was lower than that of the DM group and had no significant difference with the normal group (*p* < 0.05). Treating DM mice with Glibenclamide tablets and *A. roxburghii* can increase the content of LDL. In addition, compared with NM mice, the SOD and CAT contents of DM mice were significantly decreased, and the MDA content was significantly increased (*p* < 0.05), while DM-G, DM-Y, DM-CK, DM-P, and DM-ZP treatment groups could significantly reduce the MDA content (*p* < 0.05) and increase SOD and CAT content. It can be seen that the *A. roxburghii* under different treatments can reach values that can help maintain efficacy.

Data analysis from **Table 3** has shown that after intragastric administration with alloxan, the model group was found to significantly (*p* < 0.05) enhance blood glucose as compared to the NM group (8.533); the blood glucose level of the highest group was as high as 33.433, and the lowest was 26.933. A significant (*p* < 0.05) decrease in blood glucose of 18.2 mmol/L was observed during the concomitant treatment with Glibenclamide tablets in induced DM mice. Treatment with DM-PH, DM-CK, DM-P4, and DM-TC in induced DM mice was also shown to significantly (*p* < 0.05) reduce blood glucose by 10.6, 18.6, 18.0, and 14.9 mmol/L, respectively. However, no significant variation was observed between DM-CK and DM-P4 treatment. The mice were further dissected to observe the effect of medication or *A. roxburghii* on the organs of diabetic mice. It was found that the visceral adhesions of the mice in the DM group were more serious than those of the other groups, and the liver edge was blunt and round. Glibenclamide tablets and *A. roxburghii* could improve the above symptoms to a certain extent. The weight of the organs of mice under different treatments are shown in **Table 3**. Except for the kidney, the weight of various organs of the healthy mice in the NM group remained high. The weight of some organs (heart, lung, and pancreas) decreased in the DM group. Treating DM mice with Glibenclamide tablets for 14 days can increase the weight of some organs. Similar to DM-G, DM-P4 treatment plays a role in protecting the organs of mice.

**Table 3:** The blood glucose and organ weight (g) of mice under different treatments.

	NM	DM	DM-G	DM-PH	DM-CK	DM-P4	DM-TC
**BGBA (mmol/L)**	8.533 ± 1.650c	32.300 ± 1.300a	29.833 ± 5.093ab	26.933 ± 1.893b	33.433 ± 0.709a	31.866 ± 2.157a	29.700 ± 2.663ab
**BGAA (mmol/L)**	10.666 ± 0.321d	33.700 ± 1.552a	11.633 ± 0.709cd	16.366 ± 1.625b	14.833 ± 4.901bc	13.833 ± 1.504bcd	14.767 ± 1.858bc
**Heart**	0.264 ± 0.035a	0.157 ± 0.040c	0.187 ± 0.030bc	0.207 ± 0.043b	0.157 ± 0.025c	0.198 ± 0.041bc	0.184 ± 0.028bc
**Liver**	1.589 ± 0.342a	1.483 ± 0.386ab	1.194 ± 0.242b	1.557 ± 0.309a	1.512 ± 0.265ab	1.490 ± 0.270ab	1.609 ± 0.253a
**Spleen**	0.152 ± 0.029a	0.131 ± 0.053ab	0.088 ± 0.031ab	0.136 ± 0.030a	0.111 ± 0.054ab	0.142 ± 0.027a	0.121 ± 0.038ab
**Lung**	0.258 ± 0.043a	0.167 ± 0.051d	0.168 ± 0.073d	0.231 ± 0.030abc	0.188 ± 0.025cd	0.243 ± 0.034ab	0.208 ± 0.025bcd
**Kidney**	0.384 ± 0.081d	0.497 ± 0.11bc	0.430 ± 0.059bcd	0.525 ± 0.122ab	0.418 ± 0.064cd	0.477 ± 0.094bcd	0.620 ± 0.107a
**Pancreas**	0.119 ± 0.009ab	0.094 ± 0.008cd	0.105 ± 0.012bc	0.077 ± 0.008d	0.078 ± 0.016d	0.131 ± 0.041a	0.051 ± 0.016e
**Gonad**	0.239 ± 0.053bcd	0.175 ± 0.061cd	0.160 ± 0.069d	0.394 ± 0.174a	0.280 ± 0.034b	0.257 ± 0.051bc	0.242 ± 0.018bcd
**Thymus**	0.045 ± 0.009a	0.031 ± 0.006bc	0.026 ± 0.003c	0.030 ± 0.008bc	0.028 ± 0.006c	0.039 ± 0.014ab	0.038 ± 0.006ab
**Femur**	0.089 ± 0.005a	0.086 ± 0.004ab	0.081 ± 0.003b	0.086 ± 0.005ab	0.085 ± 0.005ab	0.084 ± 0.005ab	0.085 ± 0.005ab

BGBA, blood glucose before administration; BGAA, blood glucose after administration. Different letters indicate significant difference determined by Tukey’s test (p < 0.05; n = 10).

## Discussion

PGPB are a group of microorganisms that colonize plant roots. There has been considerable progress regarding research on different aspects of PGPB inoculants, notably the findings of more recent studies, which have revealed that PGPB are vital determinants of fertilizer savings, plant productivity, and agricultural ecosystems (Bardgett and van der Putten, 2014; Zhou et al., 2022). Some studies described that the use of PGPB can promote plant growth and enhance salt tolerance capacity by inducing synthesis of osmolytes (Chanratana et al., 2017; Karnwal, 2017). Moreover, the findings of an increasing number of studies have indicated that PGPB can modify root architecture and functioning, consequently enhancing the uptake of minerals and water to favor an increase of plant biomass (Vacheron et al., 2013; Pii et al., 2015). Overall, it has been demonstrated that PGPB inoculation has emerged as an important tool recently in the production of many crops, which are being subjected to abiotic or abiotic stresses (Mayak et al., 2004; Cordero et al., 2018; Ferreira et al., 2018).

Plant species differ in their responses to different PGPB. Our previous study demonstrated that inoculation with three PGPB (Bacillus H-15, Bacillus H-6, and Burkholderia P4) can have apositive effect on survival rate, fresh weight, polysaccharide content and decrease incidence, which resulted in significantly promoting the growth of A. roxburghii (Yang et al., 2022). On this basis, we further selected the P4 strain for further study. Firstly, this finding confirmed that the P4 strain not only can improve the survival rate, but also has a good performance in physiological indicators such as fresh weight and stem diameter of *A. roxburghii* (**Figure 1**). Furthermore, we provided an integrative understanding of crosstalks developed in plant–microorganism interrelationships for a better exploitation of PGPB in cultivation. Plant roots are one of the products in the process of plant adaptation to terrestrial life. Root activity, as an indicator of the root system capacity, seems to have three functions: biosynthesize specific components, uptake both water and nutrient, and mobilize elements in the rhizosphere soils (Peng et al., 2022). Plants with high root activity are often accompanied by higher water and nutrient assimilation capacity, and further improve biomass generation, transpiration rate, and stress tolerance in plants (Grygoruk, 2016). In our work, the root activity of *A. roxburghii* in P4 inoculation was significantly higher than that in the control, indicating that bacteria can force the plant root system to obtain resources for suitable growth. In addition, seed plants also have an important organ—leaves—for producing organic nutrients, which are the basis of plant photosynthesis and respiration. Physiological characteristics such as the size of leaf epidermal cells and the distribution of stomata are influenced by the environment including biotic and abiotic stresses. Studying the anatomical structure of leaves is a routine method to analyze their responsiveness to environmental changes and ecological adaptability (Bakker, 1991; Peguero-Pina et al., 2012). Stomata regulate gas exchange and water loss in plants. Small and dense stomata can better adapt to adversity such as strong light and drought. We found that the stomata density of *A. roxburghii* in P4 inoculation was increased, indicating that it has a strong ability to regulate water, adapt to xerophyte, and enhance its resistance to stress. Furthermore, plants will produce reactive oxygen species (ROS) with extremely active chemical properties during normal metabolism, which can react with most biological macromolecules in plants and affect the normal metabolism of cells. CAT, SOD, and POD are three important antioxidant enzymes in plants, which can scavenge harmful free radicals. Our finding showed that the P4 bacteria can improve the protective enzyme activity in *A. roxburghii* to a certain extent, resulting in improving its adaptability to the external environment.

It was well documented that plants can produce a vast array of metabolites for their evolution and adaptation to the environment (Huang et al., 2019). Another point in consideration of medicinal plant metabolites is medicinal active substance. Our previous screening has presented the dynamic changes in flavonoids, polysaccharides, and other active substances in *A. roxburghii* after different transplant times (Tao, 2019). This study further revealed the content of active substances in the roots, stems, and leaves at different times, trying to understand the effect PGPB inoculation on the accumulation and transfer of substances. The results showed that the treatment of P4 bacteria could significantly increase the content of total flavonoids in leaves and polysaccharides in stems and leaves but reduce the amount of free amino acid. As mentioned before, the flavonoids and polysaccharides in *A. roxburghii* have multiple functions such as antioxidant, anticancer, liver protection, and diabetes treatment (Zhang et al., 2007; Du et al., 2008). Free amino acids in plant tissues make up about 2% of the total nitrogen but provide a nitrogen source and a biologically active substance for plants to maintain metabolism, growth, reproduction, and immunity functions (Egydio et al., 2013). Most plants adjust nitrogen metabolism by increasing or decreasing the concentration of amino acids in the face of environmental stress (Zhang et al., 2012). Several findings also demonstrated that the higher content of amino acid in the root exudates could weaken the resistance of host plant to pathogens (Li et al., 2009; Hao et al., 2010). Thus, it can be seen that P4 bacteria could potentially improve medicinal plant quality and its ability to adapt to environmental stresses.

Further GC-MS-based metabolomics analysis was carried out to instantaneously provide not only the physiological status of samples but also an insight into the regulation of PGPB on some specialized plant metabolites as a whole, thereby understanding the regulation of PGPB on plant signaling and plant growth (Shulaev et al., 2008; Arbona et al., 2013; Hong et al., 2016). Based on multivariate statistical analysis, the results in our study showed that there were significant differences in the metabolites of *A. roxburghii* in the CK group and P4 group, in which it can be seen that the metabolism of host can respond to bacteria. A total of 19 significantly different metabolites were screened, including organic acids, amino acids, and carbohydrates. Among them, the upregulated substances included kyotorphin and piceatannol, which were vital because of their analgesic, antioxidative, and anticancer effect (Banik et al., 2020; Ueda, 2021). These results further support the importance of P4 bacteria in the accumulation of certain active substances. Furthermore, metabolic pathway analysis of differential metabolites was performed through the KEGG pathway database; among these metabolic pathways, citrate cycle, glyoxylate and dicarboxylate metabolism, and biosynthesis of unsaturated fatty acids were found to be crucial biomarker pathways contributing to plant growth after bacteria inoculation. As an important energy metabolism pathway, the citric acid cycle is the most efficient way for plants to obtain energy for various physiological activities (Schnarrenberger and Martin, 2002). Substances mapped to this metabolic pathway were upregulated, indicating that the TCA cycle of *A. roxburghii* was metabolically enhanced by probiotics. Fatty acids are essential components of biofilms and play an important role in the plant cell membrane function, as well as plant growth and development (Li et al., 2016). The proportion of unsaturated fatty acids in the membrane determines the phase transition temperature of membrane lipids. Precisely, the higher the content of unsaturated fatty acids, the stronger the membrane fluidity, thereby contributing to promoting the activity of the Na^+^/H^+^ antiporter and increasing tolerance to environmental stress (Wu et al., 2009; Sui and Han, 2014). The present study showed that substances mapped to metabolic pathways of biosynthesis of unsaturated fatty acids in this experiment were upregulated, indicating that the growth-promoting bacteria would promote the formation and accumulation of fatty acids, which results in promoting plant growth and development.

One of the main medicinal effects of *A. roxburghii* is its hypoglycemic activity (Zhang et al., 2007; Ye et al., 2017). This study investigated the efficacious effect of *A. roxburghii* in 1% alloxan-induced diabetic mice, which aimed to evaluate the scientificity and feasibility of *A. roxburghii* under P4 treatment from a safety perspective. In this study, 1% alloxan-induced diabetic mice were observed to have significant polydipsia and polyuria, which makes them a suitable diabetic model for the present study. Glibenclamide tablets are marketed as a standard drug for the symptomatic treatment of diabetic mice (Cui et al., 2013). In our study, compared with the DM group, the body weight of mice was significantly increased and the blood sugar level was significantly decreased after treatment with *A. roxburghii*, and there was no significant difference between *A. roxburghii* under different treatments and Glibenclamide, indicating that the treatment of probiotic bacteria did not reduce its hypoglycemic effect. Furthermore, the liver is the largest organ of metabolism and detoxification in the human body, and it is also the main organ for the metabolism and transformation of drugs (John, 2014). Similarly, there was no significant difference in the mouse liver between the NM group and the *A. roxburghii* treatment group, which was consistent with the results of the previous study in that *A. roxburghii* has the ability to protect the liver (Yang et al., 2017). The serum biochemical indicators and enzyme activity indicators of the mice were also the same as those of the normal group. It can be seen that the treatment effect of clematis is comparable to that of commercial drugs, and it has great application potential in the treatment of diabetes. All of the above results showed that *A. roxburghii* under P4 treatment not only had a similar effect in lowering blood sugar, but also had fewer side effects.

## Conclusion

In summary, we provided an insight into the growth-promoting effect of an exogenous rhizosphere growth-promoting bacterium on *A. roxburghii* in terms of basic inventory descriptions of plant morphology and physiology, plant metabolome, and comprehensive evaluation of pharmacological activity. Our results showed that P4 inoculation can improve stomatal density, root vigor, and POD and CAT activity of *A. roxburghii*, thereby improving plant fresh weight, growth, and survival rate. It also promoted the accumulation of polysaccharides and total flavonoids, as well as active substances kyotorphin and piceatannol, which were vital because of their analgesic effect. In addition, substances mapped to the biosynthesis of unsaturated fatty acid metabolic pathways were upregulated, which hinted at the possibility that the unsaturated fatty acid content contributed to promoting plant growth and development. Finally, the pharmacological efficacy of *A. roxburghii* was evaluated *via* a diabetic mouse model in this experiment, which showed that the *A. roxburghii* with pro-growth bacteria inoculation did not affect the hypoglycemic effect. These findings explored the high-yield and high-quality cultivation of *A. roxburghii*, providing a necessary means to enhance medicinal plant production and sustainability. However, considering these important findings of the present study, it is further suggested that the molecular mechanism of this bacterium as regards to the growth of *A. roxburghii* be explored, which can further shed light on the potential role of bacteria inoculation.

## Data availability statement

The raw data supporting the conclusions of this article will be made available by the authors, without undue reservation.

## Ethics statement

The animal study was reviewed and approved by Fujian Agriculture and Forestry University Anima Care and Use Committee.

## Author contributions

SL and WL conceived and directed the project. JW, HZ, and TC performed all of the experiments. JW and HZ performed the integrated data analysis. JW and SL wrote the manuscript. All authors contributed to the article and approved the submitted version.

## Funding

This work was supported by grants from the Natural Science Foundation of Fujian Province (2022J01592), the Putian Science and Technology Plan Project (2021NJJ005), and the Open Project of Fujian Provincial Key Laboratory of Agro-ecological Process and Safety Monitoring (NYST-2021-01).

## Conflict of interest

The authors declare that the research was conducted in the absence of any commercial or financial relationships that could be construed as a potential conflict of interest.

## Publisher’s note

All claims expressed in this article are solely those of the authors and do not necessarily represent those of their affiliated organizations, or those of the publisher, the editors and the reviewers. Any product that may be evaluated in this article, or claim that may be made by its manufacturer, is not guaranteed or endorsed by the publisher.

## References

[B1] Arbona V. Manzi M. Ollas C. Gómez-Cadenas A. (2013). Metabolomics as a tool to investigate abiotic stress tolerance in plants. Int. J. Mol. Sci. 14, 4885–4911. doi: 10.3390/ijms14034885 23455464 PMC3634444

[B2] Bais H. P. Weir T. L. Perry L. G. Gilroy S. Vivanco J. M. (2006). The role of root exudates in rhizosphere interactions with plants and other organisms. Annu. Rev. Plant Biol. 57, 233–266. doi: 10.1146/annurev.arplant.57.032905.105159 16669762

[B3] Bakker J. C. (1991). Effects of humidity on stomatal density and its relation to leaf conductance. Sci. Hortic. 48, 205–212. doi: 10.1016/0304-4238(91)90128-L

[B4] Banik K. Ranaware A. M. Choudhary H. Thakur N. Kunnumakkara A. B. (2020). Piceatannol: A natural stilbene for the prevention and treatment of cancer. Pharmacol. Res. 153, 104635. doi: 10.1016/j.phrs.2020.104635 31926274

[B5] Bardgett R. D. van der Putten W. H. (2014). Belowground biodiversity and ecosystem functioning. Nature 515, 505–511. doi: 10.1038/nature13855 25428498

[B6] Bashan Y. De-Bashan L. E. (2010). How the plant growth-promoting bacterium *Azospirillum* promotes plant growth - a critical assessment. Adv. Agron. 108, 77–136. doi: 10.1016/S0065-2113(10)08002-8

[B7] Bashan Y. de-Bashan L. E. Prabhu S. R. Hernandez J. P. (2014). Advances in plant growth-promoting bacterial inoculant technology: formulations and practical perspectives, (1998-2013). Plant Soil 378, 1–33. doi: 10.1007/s11104-013-1956-x

[B8] Chanratana M. Han G. H. Roy Choudhury A. Sundaram S. Halim M. A. Krishnamoorthy R.. (2017). Assessment of *Methylobacterim oryzae* CBMB20 aggregates for salt tolerance and plant growth promoting characteristics for bio-inoculant development. AMB Express 7, 1–10. doi: 10.1186/s13568-017-0518-7 29164352 PMC5698239

[B9] Cheng K. Wang Y. Chou H. Chang C. Lee C. Juan S. (2015). Kinsenoside-mediated lipolysis through an AMPK-dependent pathway in C3H10T1/2 adipocytes: roles of AMPK and PPARα in the lipolytic effect of kinsenoside. Phytomedicine 22, 641–647. doi: 10.1016/j.phymed.2015.04.001 26055129

[B10] Compant S. Samad A. Faist H. Sessitsch A. (2019). A review on the plant microbiome: ecology, functions, and emerging trends in microbial application. J. Adv. Res. 19, 29–37. doi: 10.1016/j.jare.2019.03.004 31341667 PMC6630030

[B11] Cordero L. Balaguer L. Rinc6n A. Pueyo J. J. (2018). Inoculation of tomato plants with selected PGPR represents a feasible alternative to chemical fertilization under salt stress. J. Plant Nutr. Soil Sci. 181, 694–703. doi: 10.1002/jpln.201700480

[B12] Costa G. Grangeia H. Figueirinha A. Figueiredo I. V. Batista M. T. (2016). Influence of harvest date and material quality on polyphenolic content and antioxidant activity of *Cymbopogon citratus* infusion. Ind. Crop Prod. 83, 738–745. doi: 10.1016/j.indcrop.2015.12.008

[B13] Creus C. M. Graziano M. Casanovas E. M. Pereyra M. A. Simontacchi M. Puntarulo S.. (2005). Nitric oxide is involved in the *Azospirillum brasilense*-induced lateral root formation in tomato. Planta 221, 297–303. doi: 10.1007/s00425-005-1523-7 15824907

[B14] Cui S. C. Yu J. Zhang X. H. Cheng M. Z. Yang L. W. Xu J. Y. (2013). Antihyperglycemic and antioxidant activity of water extract from *Anoectochilus roxburghii* in experimental diabetes. Exp. Toxicol. Pathol. 65, 485–488. doi: 10.1016/j.etp.2012.02.003 22440113

[B15] Deng Y. Q. Wei L. Zhang X. H. Yu J. (2014). Effects of polysaccharides from *Anoectochilus roxburghii* on α-glucosidase activity and blood glucose in diabetic mice. J. Shantou University 29, 41–45. doi: 1001-4217(2014)03-0041-05

[B16] Dobbelaere S. Croonenborghs A. Thys A. Vande Broek A. Vanderleyden J. (1999). Phytostimulatory effect of *Azospirillum brasilense* wild type and mutant strains altered in IAA production on wheat. Plant Soil 212, 155–164. doi: 10.1023/A:1004658000815

[B17] Dobbelaere S. Vanderleyden J. Okon Y. (2003). Plant growth-promoting effects of diazotrophs in the rhizosphere. CRC Crit. Rev. Plant Sci. 22, 107–149. doi: 10.1080/713610853

[B18] Du X. M. Nobuto I. Norihiro F. Jun H. S. Shoyama Y. H. (2008). Pharmacologically active compounds in the *Anoectochilus* and *Goodyera* species. J. Nat. Med. 62, 132–148. doi: 10.1007/s11418-007-0169-0 18404313

[B19] Egydio A. Catarina C. S. Floh E. Santos D. (2013). Free amino acid composition of *annona* (*annonaceae*) fruit species of economic interest. Ind. Crop Prod. 45, 373–376. doi: 10.1016/j.indcrop.2012.12.033

[B20] Ferreira N. C. Mavxuichelli R. D. C. L. Pacheco A. C. Araujo F. F. D. Anttunes J. E. L. Araujo A. S. F. D. (2018). *Bacillus subtilis* improves maize tolerance to salinity. Ciên. Rural. 48, e20170910. doi: 10.1590/0103-8478cr20170910

[B21] Giweli A. A. Dzamié A. M. Sokovié M. Ristié M. Janackovié P. Marin P. D. (2013). The chemical composition, antimicrobial and antioxidant activities of the essential oil of *Salvia fruticosa* growing wild in Libya. Arch. Biol. Sci. 1, 321–329. doi: 10.2298/ABS1301321G

[B22] Gouda S. Kerry R. G. Das G. Paramithiotis S. Shin H. S. Patra J. K. (2018). Revitalization of plant growth promoting rhizobacteria for sustainable development in agriculture. Microbiol. Res. 206, 131–140. doi: 10.1016/j.micres.2017.08.016 29146250

[B23] Grygoruk D. (2016). Root vitality of *Fagus sylvatica* l., *Quercus petraea* liebl. and *Acer pseudoplatanus* l. @ in mature mixed forest stand. Folia Forestalia Polonica 58, 55–61. doi: 10.1515/ffp-2016-0006

[B24] Hao W. Y. Ren L. X. Ran W. Shen Q. R. (2010). Allelopathic effects of root exudates from watermelon and rice plants on *fusarium oxysporum* f.sp. niveum. Plant Soil 336, 485–497. doi: 10.1007/s11104-010-0505-0

[B25] Hong J. Yang L. T. Zhang D. B. Shi J. X. (2016). Plant metabolomics: an indispensable system biology tool for plant science. Int. J. Mol. Sci. 17, 767. doi: 10.3390/ijms17060767 27258266 PMC4926328

[B26] Huang X. Y. Huang Y. B. Li C. Y. Zhou D. S. Chen M. J. (2017). Biomimetic wild cultivation techniques of *Anoectochilus roxburghii* under forest. Fujian Agric. Sci. Technol. 48, 41–43. doi: 10.13651/j.cnki.fjnykj.2017.05.016

[B27] Huang A. C. Jiang T. Liu Y. X. Bai Y. C. Reed J. Qu B.. (2019). A specialized metabolic network selectively modulates *Arabidopsis* root microbiota. Science 364, eaau6389. doi: 10.1126/science.aau6389 31073042

[B28] Jiang B. Zhang H. Liu C. Wang Y. Fan S. (2010). Extraction of water-soluble polysaccharide and the antioxidant activity from ginkgo biloba leaves. Med. Che Res. 19, 262–270. doi: 10.1007/s00044-009-9189-5

[B29] Jin X. Wang Z. Wu F. Li X. Zhou X. (2022). Litter mixing alters microbial decomposer community to accelerate tomato root litter decomposition. Microbiol. Spect 10, e0018622. doi: 10.1128/spectrum.00186-22 PMC9241821 35604181

[B30] John Y. L. C. (2014). Liver physiology: metabolism and detoxification. Pathophysiol. Hum. Dis., 1770–1782. doi: 10.1016/B978-0-12-386456-7.04202-7

[B31] Karnwal A. (2017). Isolation and identification of plant growth promoting rhizobacteria from maize (ea mays l.) rhizosphere and their plant growth promoting effect on rice (*Oryza sativa* l). J. Plant Prot. Res. 57, 144–151. doi: 10.1515/JPPR-2017-0020

[B32] Li X. G. Liu B. Heia S. Liu D. D. Han Z. M. Zhou K. X.. (2009). The effect of root exudates from two transgenic insect-resistant cotton lines on the growth of *Fusarium oxysporum* . Transgenic Res. 18, 757–767. doi: 10.1007/s11248-009-9264-1 19396562

[B33] Li N. Xu C. Li-Beisson Y. Philippar K. (2016). Fatty acid and lipid transport in plant cells. Trends Plant Sci. 21, 145–158. doi: 10.1016/j.tplants.2015.10.011 26616197

[B34] Mahdavikia F. Saharkhiz M. J. Karami A. (2017). Defensive response of radish seedlings to the oxidative stress arising from phenolic compounds in the extract of peppermint (*Mentha x piperita* l.). Sci. Hortic. 214, 133–140. doi: 10.1016/j.scienta.2016.11.029

[B35] Mayak S. Tirosh T. GHick B. R. (2004). Plant growth-promoting bacteria confer resistance in tomato plants to salt stress. Plant Physiol. Biochem. 42, 565–572. doi: 10.1016/j.plaphy.2004.05.009 15246071

[B36] Molina-Favero C. Creus C. M. Simontacchi M. Puntarulo S. Lamattina L. (2008). Aerobic nitric oxide production by *Azospirillum brasilense* Sp245 and its influence on root architecture in tomato. Mol. Plant Microbe in. 21, 1001–1009. doi: 10.1094/MPMI-21-7-1001 18533840

[B37] Niu H. Xie Z. M. Li G. U. Liang Y. Wei K. H. Wang J. M.. (2018). Effects of planting density and harvesting stages for *anoectochilus roxburghii* planted under forest on its yield and quality. Modern Chin. Med. 20, 837–841+865. doi: 10.13313/j.issn.1673-4890.20180302005

[B38] Peguero-Pina J. J. Flexas J. Galmés J. NIINEMETS Ü. L. O. Sancho-Knapik D. Barredo G.. (2012). Leaf anatomical properties in relation to differences in mesophyll conductance to CO2 and photosynthesis in two related *Mediterranean abies* species. Plant Cell Environ. 35, 2121–2129. doi: 10.1111/j.1365-3040.2012.02540.x 22594917

[B39] Peng X. Li J. Sun L. Gao Y. Cao M. Luo J. (2022). Impacts of water deficit and post-drought irrigation on transpiration rate, root activity, and biomass yield of *festuca arundinacea* during phytoextraction. Chemosphere 294, 133842. doi: 10.1016/j.chemosphere.2022.133842 35120948

[B40] Pii Y. Mimmo T. Tomasi N. Terzano R. Cesco S. Crecchio C. (2015). Microbial interactions in the rhizosphere: beneficial influences of plant growth-promoting rhizobacteria on nutrient acquisition process. a review. Biol. Fert. Soils. 51, 403–415. doi: 10.1007/s00374-015-0996-1

[B41] Qin P. (2013). The analysis of the genetic diversity and effective components of anoectochilus (Fuzhou, China: Fujian Agriculture and Forestry University).

[B42] Rout M. E. Southworth D. (2013). The root microbiome influences scales from molecules to ecosystems: the unseen majority. Am. J. Bot. 100, 1689–1691. doi: 10.3732/ajb.1300291 24008514

[B43] Rudrappa T. Kirk J. Czymmek P. W. Paré P. W. Bais H. P. (2008). Root-secreted malic acid recruits beneficial soil bacteria. Plant Physiol. 148, 1547–1556. doi: 10.1104/pp.108.127613 18820082 PMC2577262

[B44] Sampaio B. L. Edrada-Ebel R. A. Da Costa F. B. (2016). Effect of the environment on the secondary metabolic profile of *tithonia diversifolia*: a model for environmental metabolomics of plants. Sci. Rep. 6, 29265. doi: 10.1038/srep29265 27383265 PMC4935878

[B45] Santos C. L. R. Alves G. C. de Matos Macedo A. V. Giori E. G. Pereira W. Urquiaga S.. (2017). Contribution of a mixed inoculant containing strains of burkholderia spp. and *Herbaspirillum* ssp. to the growth of three sorghum genotypes under increased nitrogen fertilization levels. Appl. Soil Ecol. 113, 96–106. doi: 10.1016/j.apsoil.2017.02.008

[B46] Schnarrenberger C. Martin W. (2002). Evolution of the enzymes of the citric acid cycle and the glyoxylate cycle of higher plants: a case study of endosymbiotic gene transfer. Eur. J. Biochem. 269, 868–883. doi: 10.1046/j.0014-2956.2001.02722.x 11846788

[B47] Shao Q. S. Deng Y. M. Liu H. B. Zhang A. L. Huang Y. Q. Xu G. Z.. (2014a). Essential oils extraction from *Anoectochilus roxburghi* using supercritical carbon dioxide and their antioxidant activity. Ind. Crop Prod. 60, 104–112. doi: 10.1016/j.indcrop.2014.06.009

[B48] Shao Q. S. Wang H. Z. Guo H. P. Zhou A. C. Huang Y. Q. Sun Y. L.. (2014b). Effects of shade treatments on photosynthetic characteristics, chloroplast ultrastructure, and physiology of *Anoectochilus roxburghii* . PloS One 9, e85996. doi: 10.1371/journal.pone.0085996 24516523 PMC3917826

[B49] Shulaev V. Cortes D. Miller G. Mittler R. (2008). Metabolomics for plant stress response. Physiol. Plantarum. 132, 199–208. doi: 10.1111/j.1399-3054.2007.01025.x 18251861

[B50] Song Z. P. Jin R. M. Fu S. G. Zhang W. B. Li X. (2007). Action of lowering blood glucose of tissue cultured- seedlings extract of *Anoectochilus roxburghii* on diabetic mice. SH J. TCM 41, 74–75. doi: 10.16305/j.1007-1334.2007.01.032

[B51] Souza D. R. Ambrosini A. A. Passaglia L. (2015). Plant growth-promoting bacteria as inoculants in agricultural soils. Genet. Mol. Biol. 38, 401–419. doi: 10.1590/S1415-475738420150053 26537605 PMC4763327

[B52] Sui N. Han G. H. (2014). Increases of unsaturated fatty acids in membrane lipids protects photosystem II from photoinhibition under salinity in different halophytes. J. Agric. Sci. 6, 251. doi: 10.5539/jas.v6n12p251

[B53] Tang T. T. Duan X. Y. Ke Y. Zhang L. Shen Y. B. Hu B.. (2018). Antidiabetic activities of polysaccharides from *anoectochilus roxburghii* and *anoectochilus formosanus* in STZ-induced diabetic mice. Int. J. Biol. Macromol. 112, 882–888. doi: 10.1016/j.ijbiomac.2018.02.042 29438753

[B54] Tao Z. X. (2019). Study on the dynamic change of quality of wild-imitated *Anoectochilus roxburghii* cultivated under forest. (Fuzhou, China: Fujian Agric. Forestry University).

[B55] Ueda H. (2021). Review of kyotorphin research: a mysterious opioid analgesic dipeptide and its molecular, physiological, and pharmacological characteristics. Front. Med. Technol. 3. doi: 10.3389/fmedt.2021.662697 PMC8757751 35047919

[B56] Vacheron J. Desbrosses G. Bouffaud M. L. Touraine B. Moenne-Loccoz Y. Muller D.. (2013). Plant growth-promoting rhizobacteria and root system functioning. Front. Plant Sci. 4. doi: 10.3389/fpls.2013.00356 PMC3775148 24062756

[B57] Van Loon L. C. (2007). Plant responses to plant growth-promoting bacteria. Eur. J. Plant Pathol. 119, 243–254. doi: 10.1007/s10658-007-9165-1

[B58] Wang L. P. Chen Q. W. Zhuang S. Q. Wen Y. Y. Cheng W. Q. Zeng Z. J.. (2020). Effect of *Anoectochilus roxburghii* flavonoids extract on H_2_O_2_ - induced oxidative stress in LO_2_ cells and d-gal induced aging mice model. J. Ethnopharmacol. 254, 112670. doi: 10.1016/j.jep.2020.112670 32135242

[B59] Weckwerth W. Wenzel K. Fiehn O. (2004). Process for the integrated extraction, identification and quantification of metabolites, proteins and RNA to reveal their co-regulation in biochemical networks. Proteomics 4, 78–83. doi: 10.1002/pmic.200200500 14730673

[B60] Wen B. Mei Z. L. Liu S. Q. (2017). metaX: a flexible and comprehensive software for processing metabolomics data. BMC Bioinf. 18, 183. doi: 10.1186/s12859-017-1579-y PMC5361702 28327092

[B61] Wu L. K. Chen J. Lin W. X. Wu H. M. Lin S. (2019). An antagonistic antibacterial strain for preventing and controlling root rot of pseudostellaria heterophylla and its application. (State Intellectual Property Office of the People's Republic of China).

[B62] Wu Q. Liu T. Liu H. Zheng G. C. (2009). Unsaturated fatty acid: metabolism, synthesis and gene regulation. Afr J. Biotechnol. 8 (9), 1782–1785. doi: 10.4314/AJB.V8I9.60381

[B63] Yang Z. G. Zhang X. H. Yang L. W. Pang Q. Y. Li J. Wu Y. F.. (2017). Protective effect of *anoectochilus roxburghii* polysaccharide against CCl_4_-induced oxidative liver damage in mice. Int. J. Biol. Macromol. 96, 442–450. doi: 10.1016/j.ijbiomac.2016.12.039 27993656

[B64] Yang J. Zhao H. Y. Chen T. Zhou X. Y. Lin W. X. Lin S. (2022). Effects of different rhizosphere promoting bacteria on physiological characteristics of *Anoectochilus roxburghii* . J. Anhui Agric. Sci. 50, 186–189. doi: 10.3969/j.issn.0517-6611.2022.02.051

[B65] Ye S. Y. Shao Q. S. Zhang A. L. (2017). *Anoectochilus roxburghii*: A review of its phytochemistry, pharmacology, and clinical applications. J. Ethnopharmacol. 209, 184–202. doi: 10.1016/j.jep.2017.07.032 28755972

[B66] Yu X. L. Lin S. E. Zhang J. Q. Huang L. Y. Yao H. (2017). Purification of polysaccharide from artificially cultivated *anoectochilus roxburghii* (wall.) lindl. by high-speed counter current chromatography and its antitumor activity. J. Sep Sci. 40, 4338–4346. doi: 10.1002/jssc.201700340 28892307

[B67] Yu S. J. Shi Z. Q. (2016). Ratio test of growing media on *Anoectochilus formosanus* . Modern Agric. Technol. 13, 86–87. doi: 1007-5739(2016)13-0086-02

[B68] Zhang Y. Cai J. Ruan H. Pi H. Wu J. (2007). Antihyperglycemic activity of kinsenoside, a high yielding constituent from *Anoectochilus roxburghii* in streptozotocin diabetic rats. J. Ethnopharmacol. 114, 141–145. doi: 10.1016/j.jep.2007.05.022 17869039

[B69] Zhang P. Fu J. Hu L. (2012). Effects of alkali stress on growth, free amino acids and carbohydrates metabolism in kentucky bluegrass (*poa pratensis*). Ecotoxicology 21, 1911–1918. doi: 10.1007/s10646-012-0924-1 22592662

[B70] Zhang X. Huang G. Bian X. Zhao Q. (2013). Effects of root interaction and nitrogen fertilization on the chlorophyll content, root activity, photosynthetic characteristics of intercropped soybean and microbial quantity in the rhizosphere. Plant Soil Environ. 59, 80–88. doi: 10.1007/s11104-012-1528-5

[B71] Zhou X. Zhang X. Ma C. Wu F. Jin X. Dini-Andreote F.. (2022). Biochar amendment reduces cadmium uptake by stimulating cadmium-resistant PGPR in tomato rhizosphere. Chemosphere 307, 136138. doi: 10.1016/j.chemosphere.2022.136138 36002065

[B72] Zhu H. Wang Y. Liu Y. Xia Y. Tang T. (2009). Analysis of flavonoids in *Portulaca oleracea* l. by UV-vis spectrophotometry with comparative study on different extraction technologies. Food Anal. Methods 3, 90–97. doi: 10.1007/s12161-009-9091-2

